# Occupational exposures to blood and body fluids among healthcare workers in Ethiopia: a systematic review and meta-analysis

**DOI:** 10.1186/s12199-020-00897-y

**Published:** 2020-10-03

**Authors:** Biniyam Sahiledengle, Yohannes Tekalegn, Demelash Woldeyohannes, Bruce John Edward Quisido

**Affiliations:** 1 Department of Public Health, Madda Walabu University Goba Referral Hospital, P.O. Box: 76, Bale Goba, Ethiopia; 2 Department of Public Health, College of Medicine and Health Science, Wachemo University, Hosanna, Ethiopia; 3 Department of Nursing, College of Health Science, Madda Walabu University Goba Referral Hospital, Bale-Goba, Ethiopia

**Keywords:** Blood and body fluid, Ethiopia, Healthcare workers, Mucocutaneous exposure, Occupational exposure, Splash

## Abstract

**Background:**

Occupational exposure to blood and body fluids is a major risk factor for the transmission of blood-borne infections to healthcare workers. There are several primary studies in Ethiopia yet they might not be at the national level to quantify the extent of occupational blood and body fluid exposures (splash of blood or other body fluids into the eyes, nose, or mouth) or blood contact with non-intact skin among the healthcare workers. This systematic review and meta-analysis aimed to estimate the pooled prevalence of occupational blood and body fluid exposure of healthcare workers in Ethiopia.

**Methods:**

PubMed, Science Direct, Hinari, Google Scholar, and the Cochrane library were systematically searched; withal, the references of appended articles were also checked for further possible sources. The Cochrane *Q* test statistics and *I*^2^ tests were used to assess the heterogeneity of the included studies. A random-effects meta-analysis model was used to estimate the lifetime and 12-month prevalence of occupational exposure to blood and body fluids among healthcare workers in Ethiopia.

**Results:**

Of the 641 articles identified through the database search, 36 studies were included in the final analysis. The estimated pooled lifetime and 12-month prevalence on occupational exposure to blood and body fluids among healthcare workers were found to be at 54.95% (95% confidence interval (CI), 48.25–61.65) and 44.24% (95% CI, 36.98-51.51), respectively. The study identified a variation in healthcare workers who were exposed to blood and body fluids across Ethiopian regions.

**Conclusion:**

The finding of the present study revealed that there was a high level of annual and lifetime exposures to blood and body fluids among healthcare workers in Ethiopia.

## Introduction

Occupational exposure to blood and body fluids (BBFs) is a major risk factor for the transmission of blood-borne infections to healthcare workers (HCWs). These exposures can heighten the risk of infection to human immunodeficiency virus (HIV), hepatitis B, and hepatitis C. In many cases, exposures occur through mucocutaneous injury (splash of blood or other body fluids into the eyes, nose, or mouth) or non-intact skin exposure, and percutaneous injury (occurs as a result of a break in the skin caused by a needle stick or sharps contaminated with blood or body fluids) [1–5].

According to the World Health Organization (WHO), it is estimated that about 3 million HCWs are exposed to bloodborne pathogens each year—occupational exposure causes approximately 170,000 to HIV infections, 2 million to HBV infections, and 0.9 million to HCV infections [3]. A recent review stipulated that the prevalence of infections, such as HCV is significantly higher in HCWs than in the general population [1]. A review by Tavoschi et al. in the European Union/European Economic Area also indicate high levels of HBV and HCV infection among specific groups (such as HCWs); estimates varied widely from 0.4 to 11.7% for HBV and from 0.7 to over 90% for HCV with most being higher than in the general population [4].

Numerous systematic reviews on BBFs exposure, prevalence of blood-borne infections, and the determinant factors were explored so far [1, 4, 6–9]. For instant, a review by Auta et al. has demonstrated that HCWs who had received training on infection prevention and occupational exposure to blood and body fluids. The risk of occupational exposure in the preceding 12 months among healthcare workers without training was significantly higher than in trained staff (RR, 1.79, 95% CI, 1.23–2.07) [9]. Another review identified reasons for occupational BBFs: a sudden movement of the patient during blood sampling; during childbirth; during the handling of specimens; due to a lack of Personal Protective Equipment (PPE), experience years of HCWs, and working for more than 40 h/week [7].

Despite previous efforts, there are limited data about the extent of mucocutaneous injury and its driving forces particularly in the developing world [7–9]. Of note, antecedent literatures publicized that the face is the most common exposure site reported of mucocutaneous injury: healthcare workers’ eyes (conjunctiva) were exposed to BBF (53%) of all reported cases. The mucosa of the mouth and nose were exposed in 11% and 5% of cases, respectively [5]. Prevalence studies also have revealed that a high level of splashes of fluids to an extent inevitable among HCWs than any percutaneous injury in low-income settings [10, 11].

In Sub-Saharan Africa, HCWs are at a consequential risk of infection from blood-borne pathogens because of the excessive prevalence of such blood-borne infections in the general population [8, 9]. A systematic review conducted in 21 African countries found a high prevalence of occupational exposures to blood and body fluids among HCWs—about two-thirds were exposed during their entire career, and almost half of them were exposed each year [9]. Additionally, evidence from every region of Africa indicates considerable variations in the prevalence of blood and body fluid exposures. The 12-month prevalence of all the types of mucocutaneous injury ranged from 17.0 to 67.6% in Kenyan and Burundian studies. The estimated pooled 12-month prevalence was 48.0%. Regional pooled estimates covered from 33.9 to 60.7% in Southern Africa and Northern Africa [9].

In Ethiopia, occupational exposure to BBFs is a pressing concern and continues to have a significant problem in its healthcare system [10–14]. Antecedent studies also reported that standard precaution practices among HCWs were suboptimal, and the lack of compliance with these measures is still a great lookout [11, 13, 15, 16]. Though attention is paid to the safety of HCWs through the National Infection Prevention and Patient Safety (IPPS) initiatives, the number of exposures to BBFs reported did not manifest a sign of decline as evidenced by some studies [11, 14, 15, 17]. Several primary studies in Ethiopia conveyed a high prevalence of mucocutaneous injury. However, the results were inconsistent [11, 16–23].. For some instances, in Central Ethiopia, the prevalence of a 12-month splash of blood or other body fluids into the eyes, nose, or mouth exposures among HCWs was 19.9% [11] and 41.3% [20]; in North Ethiopia 60.2% [16] and 31.7% [21]; and in East Ethiopia 43.8% [18] and 20.2% [14]. Currently in Ethiopia, no report exists to quantify the pooled prevalence of mucocutaneous injury among HCWs; even the existing review determined the prevalence of needle stick injury and did not estimate mucocutaneous injury [24]. Moreover, poor compliance toward standard precautions and inadequate infection prevention knowledge seemed to be common among HCWs, reflecting a potentially risk of BBFs exposure at healthcare facilities in Ethiopia [10, 12, 13]. Given these developments, it is timely and crucial to investigate the burden of occupational BBF exposures among HCWs. Therefore, the objective of the present systematic review and meta-analysis directs to estimate the pooled prevalence of BBFs among HCWs in Ethiopia.

## Methods

This systematic review and meta-analysis were conducted subsequent to “the Preferred Reporting Items for Systematic Reviews and Meta-Analyses (PRISMA)” guidelines [25]. Studies were favored according to the criteria outlined below (Additional file 1).

### Eligibility criteria

#### Study designs

In this review, we appended cross-sectional studies and baseline assessment of longitudinal studies. Studies that reported the lifetime and/or 12-month prevalence of occupational exposure through blood and/or body fluid exposures to mucous membranes and broken skins were eligible to be included in the present review. Systematic reviews, letters to editors, short communications, qualitative studies, case series, case-control studies, and case reports were excluded. Also, articles that were not fully accessible, unsuccessful two-email contacts with the primary/corresponding authors were excluded, too. In addition to the aforementioned, studies restricted to HCWs’ needle stick and/or sharp injuries were excluded when data were not provided separately for blood and body fluid exposures. Lastly, the aggregate reports for blood and/or body fluid exposures and needle stick and/or sharp injuries were debarred from the study.

#### Participants

Studies who met the following criteria were considered for inclusion:

##### Population

Healthcare works (HCWs) with direct contact to patients or blood/body fluids. We also encompassed studies, which were conducted on a specific segment of the healthcare workforce (such as physicians, nurses, midwives, laboratory technicians, and cleaners).

##### Exposure

Study examines occupational BBFs exposures.

#### Study period

No restriction on publication date, since there is no prior study in the country that examines occupational BBFs exposures.

#### Language

Articles which were only reported in the English language.

### Article searching strategy

MEDLINE/PubMed, Hinari, Science Direct, and the Cochrane Library databases from inception until January 31, 2020, that reported the prevalence of occupational exposures to blood and/or body fluids among HCWs in Ethiopia were sought. Literature search strategies were developed using medical subject headings (MeSH) and text words related to occupational blood and/or body fluid exposures. The following search terms were used and combined using Boolean operators: “prevalence”, “magnitude”, “occupation”, “exposure”, “accident”, “occupational exposure”, “accidental exposure”, “accidental occupational exposure”, “occupational disease”, “occupational hazard”, “cross-infection”, “blood”, “body fluid”, “blood spill”, “blood-borne pathogens”, “blood-borne infection”, “health-care workers”, “health workers”, “medical personnel”, “health personnel”, and “Ethiopia”. The electronic database search was also supplemented by searching for gray literature through Google scholar, Google searching, and Ethiopian University digital repositories (such as the Addis Ababa University Digital Library). To ensure literature saturation, the reference list of appended studies and/or relevant studies identified through the search were scanned as well. Finally, the literature search was limited to the English language and human subjects (Additional file 2).

### Operational definition

#### Occupational blood and body fluid exposure

In this review, “occupational blood and body fluid exposure” indicates mucocutaneous exposure. Mucocutaneous exposure is defined as any exposure to blood or body fluid splashes into the eyes, nose, or mouth or blood contact with non-intact skin. We appended studies that reported the lifetime or 12-month prevalence of occupational exposure through blood and body fluid contacts from at least one of these routes (eye, mouth, mucous membrane, and non-intact skin).

#### Healthcare workers

Healthcare workers (HCWs) are referred to as paid or unpaid individuals (e.g., full-time employees or medical students) working in a healthcare setting whose activities involve direct contact with patients, or with blood or other body fluids from the patients. Hence, we incorporated studies, which involved physicians, nurses, midwives, health officers, laboratory technicians, anesthetists, auxiliary healthcare workers, residents, or interns undertaking clinical training or gaining experiences in the healthcare settings.

### Study selection and data extraction

In this review, all the searched articles were imported into the EndNote version X^4^ software, and after that, the duplicate articles were removed. Two investigators (BS and YT) independently screened and identified articles by their titles, abstracts, and full-texts to determine eligibilities against predetermined inclusion and exclusion criteria. Afterward, the screened articles were compiled together by the two investigators, and discrepancies were resolved through unanimous consensus.

The data extraction form was prepared using Microsoft Excel spreadsheet. Two reviewers extracted data from the studies and were entered into Microsoft Excel. The data extraction form included (i) name of the primary author; (ii) year of publication; (iii) region; (iv) sample size; (v) study population; (vi) type of study design; (vii) sampling technique (viii) response rate; and (ix) 12 months and lifetime prevalence of blood and body fluid exposure among HCWs.

### Quality assessment

The qualities of the appended studies were assessed and the risks for biases were judged using the Joanna Briggs Institute (JBI) quality assessment tool for the prevalence studies [26]. There were nine parameters: (1) appropriate sampling frame, (2) proper sampling technique, (3) adequate sample size, (4) study subject and setting description, (5) sufficient data analysis, (6) use of valid methods for the identified conditions, (7) valid measurement for all participants, (8) using appropriate statistical analysis, and (9) adequate response rate (adequate if 60% or higher). Failure to satisfy each parameter was scored as 1 if not 0. The risks for biases were classified as either low (total score, 0 to 2), moderate (total score, 3 or 4), or high (total score, 5 to 9). Two reviewers (BS and YT) assessed the quality of the studies included. Finally, articles with scores of 5 to 9, which meant having a high risk of biases were debarred (Additional file 3).

### Statistical analysis

Primarily, appended studies were categorized whether they have measured the lifetime prevalence of blood and body fluid exposures or whether they are on a 12-month prevalence, and later were entered into the STATA version 14. The meta pop program was utilized to estimate the pooled prevalence of lifetime and 12-month prevalence of blood and body fluid exposure among HCWs. Accordingly, the prevalence of blood and body fluid exposure (*p*) were estimated using data from the appended studies which reported the proportion of HCWs who were exposed to body fluids at any time during their career, and 12-month prevalence was appraised using data from the studies which reported the proportion of participants exposed to body fluids in the preceding 12 months. Corresponding standard errors (SE) were calculated using se = √*p*(1−*p*)/*n*. The researchers estimated the pooled prevalence of blood and body fluid exposures using random-effects meta-analysis based on DerSimonian and Laird approach. The existence of heterogeneity among the studies was checked using the *I*^2^ test statistics. Heterogeneity will be classified into the following three categories: low heterogeneity (*I*^2^ index < 25%), average heterogeneity (*I*^2^ index = 25–75%), and high heterogeneity (*I*^2^ index > 75%). Also, a *p* value of < 0.05 is used to declare heterogeneity. Thus, a random-effects model was used to analyze data in this study, since the estimated both 12 months and lifetime prevalence of BBFs was found to be high. Finally, meta-regression analysis was used to evaluate the association between the prevalence of BBFs and publication year, and sample size in the selected studies. We utilized STATA version 14 statistical software (StataCorp LP.2015, College Station, TX: USA) for all statistical analyses.

### Publication bias

In this meta-analysis, possible publication biases were visualized thru funnel plots. Symmetrical large inverted funnels resembled the absence of publication biases. Also, the probability of publication biases were tested using two main statistical methods (Egger’s and Begg’s tests) which were wielded to test funnel plot asymmetries. The level of significance for asymmetries was viewed as *p* < 0.05.

### Sensitivity analysis

Also, sensitivity analyses were undertaken—the stability of the pooled estimate for each study. The investigation was done by excluding a single individual study from the analysis at a time to explore the robustness of the findings.

## Results

### Description of the studies

**Fig. 1:**
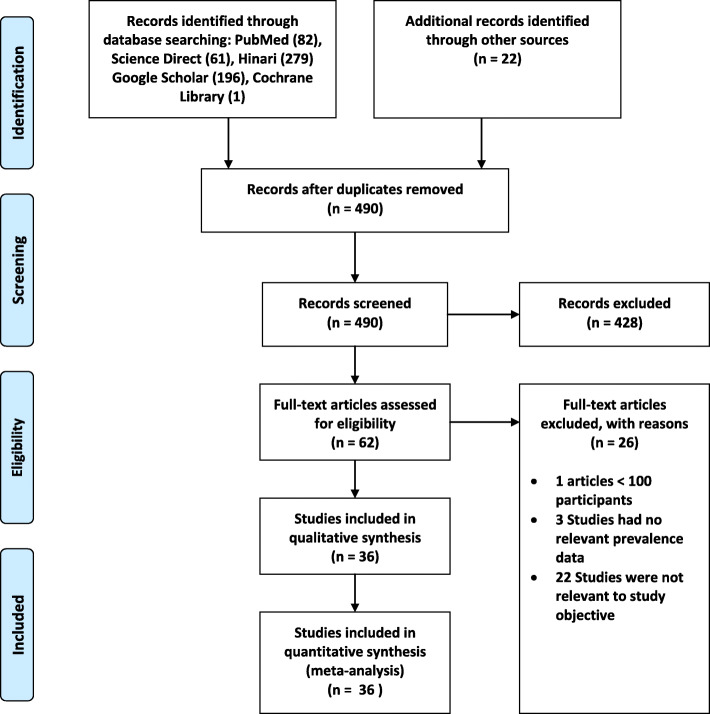
Flow diagram, systematic review, blood and body fluid exposure among healthcare workers in Ethiopia, 2007–2020

The initial electronic searches generated 641 studies using international databases and Ethiopian University research repositories. The database included PubMed (82), Science Direct (61), Hinari (279), Google Scholar (196), Cochrane Library (1), and the remaining 22 studies were identified through manual search. Of these, 151 duplicates were identified and effaced. From the tarry of 490 articles, based on the pre-defined eligibility criteria, 428 articles were excluded after reading their titles and abstracts. Sixty-two full-text articles remained and were further assessed for their eligibilities. Finally, based on the pre-defined inclusion and exclusion criteria and quality assessment, only 36 articles were extracted for the final analyses [10–14, 16–23, 27–49] (Fig. 1).

### Characteristics of the appended studies

The general characteristics of the favored articles were presented in Table 1. Of the 36 articles included in this review and meta-analyses, 14 were conducted in Addis Ababa; 9 in the Amhara Region; 6 in Oromia Region; 4 from the Southern Nations, Nationalities, and People (SNNP); 2 in Harari Region; and only 1 from Tigray Region. A total number of 10,973 healthcare workers participated in the study—the highest and lowest sample sizes were from the studies of Geberemariyam et al. [13] in the Oromia Region (648 HCWs), and [46] in Addis Ababa (104 HCWs). All the appended studies were cross-sectional studies. Twenty-two studies were conducted solely among hospital healthcare workers. Among the studies, twenty-three of them also presented data regarding 12-month prevalence on occupational exposures to BBFs [10–12, 14, 16, 18–23, 27–29, 35, 37–39, 43–46, 48],,, and the lifetime prevalence on BBF exposures were reported in twenty-five studies [11–15, 17, 19, 20, 22, 28–34, 36, 39–42, 44, 45, 47, 49].. From the studies, thirteen articles have reported having both the 12-month and lifetime BBFs exposure prevalence [10–12, 14, 19, 20, 22, 28, 29, 39, 43–45].The latest article was published in 2020 [10], and the earliest study was concluded last 2007 [44]. The prevalence of 12 months BBFs among the Ethiopian HCWs ranged from 16.5 [12] to 67.5% [23] in Addis Ababa Region. The lifetime prevalence of BBFs varied from 28.8% in the Harari Region [14] to 81.0% in the Amhara Region [33]. In this review, a low risk of bias was realized in 32 (88.9%) of the included studies (Additional file 3).

**Table 1:** Studies identified in the systematic review on blood and body fluid exposure among healthcare workers in Ethiopia, 2007–2020

Name	Year of publication	Study design	Study population	Setting	Sampling	Region	Sample size	Response rate	12-month prevalence of BBE exposure	Life time prevalence of BBE exposure	Type of BBF exposure	Risk of bias
Zenbaba D et al. [10]	2020	CS	HCWs and C	Hospitals	Simple random	Oromia	394	97.5	44.9	60.2	E, M or MM	L
Geberemariyam BS et al. [13]	2018	CS	HCWs	Hospitals and health centers	Simple random	Oromia	648	95.3		39	E or M	L
Reda AA et al. [14]	2010	CS	HCWs	Hospitals and health centers	Census	Harari and Dire Dawa	484	84.4	20.2	28.8	E or M	L
Geberemariyam BS [11]	2019	CS	HCWs	Hospitals	Simple random	Addis Ababa	277	85.7	29.2	42.6	E or M	L
Amare Z et al. [27]	2018	CS	HCWs	Hospital	Simple random	Addis Ababa	200	100	38		E or M	L
Gebresilassie A et al. [16]	2014	CS	HCWs	Hospitals and health centers	Simple random	Tigray	483	95.6	60.2		E, M or N	L
Kaweti and Abegaz [22]	2014	CS	HCWs	Hospitals	Simple random	SNNP	496	94.3	46	28	MM	L
Amerga and Mekonnen [20]	2018	CS	HCWs	Health centers	Simple random	Addis Ababa	361	93.2	40.2	47.4	NR	L
Tadesse M et al. [28]	2016	CS	HCWs	Hospitals and health centers	Systematic sampling	SNNP	623	82	65.7	73.8	NR	L
Yenesew and Fekadu [29]	2014	CS	HCWs	Hospitals and health centers	Simple random	Amhara	317	95	65.9	76	MM	L
Yakob E et al. [30]	2015	CS	HCWs	Hospital	Census	SNNP	135	93.8		45.2	E or M	L
Mengesha and Yirsaw [32]	2014	CS	HCWs	Hospitals and health centers	Census	SNNP	162	73.3		70.4	E or M	L
Asmr Y et al. [32]	2019	CS	HCWs	Hospitals	Simple random	Addis Ababa	123	96.1		36.6	E, M or N	L
Gebremariam AA et al. [33]	2019	CS	HCWs	Hospital	Census	Amhara	332	100		81	MM	L
Desalegn Z et al. [34]	2015	CS	HCWs	Hospitals	Convenience	Addis Ababa	254	100		72.8	E or M	M
Jemaneh L [35]	2014	CS	HCWs	Hospital	Convenience	Addis Ababa	146	97.9	27.9		NR	M
Desta B [36]	2017	CS	HCWs	Hospital	Convenience	Addis Ababa	142	90.4		57	E, M or N	M
Tesfay and Habtewold [37]	2014	CS	HCWs	Hospitals and health centers	Stratified sampling technique	Amhara	234	90.2	56.7		MM	L
Beyera and Beyen [21]	2014	CS	HCWs	Hospitals and health centers	Simple random	Amhara	401	95	40.4		MM	L
Yallew WW [38]	2017	CS	HCWs	Hospitals	Simple random	Amhara	413	97.8	56.7		E or M	L
Hebo HJ et al. [39]	2019	CS	HCWs	Hospital	Simple random	Oromia	230	95.8	43	60	E or M	L
Yasin J et al. [19]	2019	CS	HCWs	Hospital	Stratified sampling technique	Amhara	282	100	39	58.5	E or M	L
Alemayehu T et al. [18]	2016	CS	C	Hospitals and health centers	Multistage sampling	Harari	250	98.8	43.8		NR	L
Abeje and Azage [40]	2015	CS	HCWs	Hospitals and health centers	Simple random	Amhara	370	98.9		69.2	E or M	L
Sahiledengle B et al. [12]	2018	CS	HCWs	Hospitals and health centers	Stratified sampling technique	Addis Ababa	605	96.2	16.5	39.8	E or M	L
Tebeje and Hailu [41]	2010	CS	HCWs	Hospitals and health centers	Stratified sampling technique	Oromia	254	95.8		57.1	MM	L
Akalu GT et al. [42]	2016	CS	HCWs and C	Hospital	Convenience	Addis Ababa	313	100		57.2	E or M	M
Yimechew Z et al. [43]	2013	CS	HCWs and C	Hospital	Stratified sampling technique	Amhara	252	88.4	62.3	70.2	MM	L
Belachew YB et al. [17]	2017	CS	HCWs	Hospitals	Census	Oromia	318	93.3		62.6	MM	L
Damta M [44]	2007	CS	HCWs and C	Hospitals and health centers	Simple random	Amhara	351	93.4	20.2	45.8	MM	L
Atlaw WD [45]	2013	CS	HCWs	Hospital	Stratified sampling technique	Addis Ababa	290	87.3	33.5	66.5	E	L
Gebreselassie FT [46]	2009	CS	HCWs	Hospital	Simple random	Addis Ababa	104	98.1	67.3		E, M or MM	L
Abreha N [47]	2018	CS	HCWs	Hospital	Convenience	Addis Ababa	108	94.4		56.9	MM	M
Girmaye E et al. [48]	2018	CS	HCWs and C	Hospital	Systematic sampling	Addis Ababa	244	100	34.4		E, M or N	L
Shiferaw Y et al. [23]	2012	CS	C	Hospitals	Census	Addis Ababa	126	100	67.5		MM	L
Alemu B [49]	2014	CS	HCWs and C	Hospital	Convenience	Oromia	251	100		41	E or M	L

*L* low risk of bias, *M* moderate risk of bias, *HCWs* healthcare workers, *C* cleaners/waste handlers, *CS* cross-sectional study design, *E* splashing of blood or body fluids into the eyes, *M* splashing of blood or body fluids into the mouth, *N* splashing of blood or body fluids into the nose, *MM* splashing of blood or body fluids to any mucous membranes, *NR* not reported

### Prevalence of blood and body fluid exposures among HCWs in Ethiopia

**Fig. 2:**
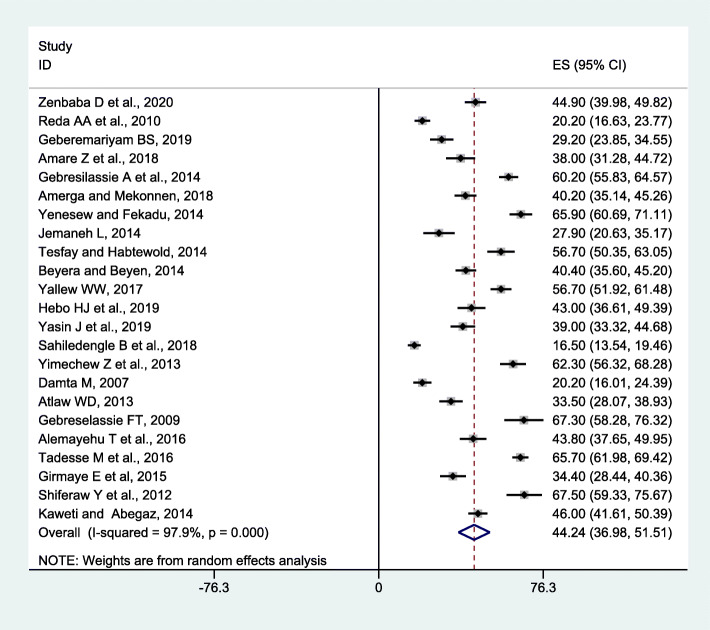
Meta-analysis, 12-month prevalence of blood and body fluid exposure among healthcare workers in Ethiopia, 2007–2020

**Fig. 3:**
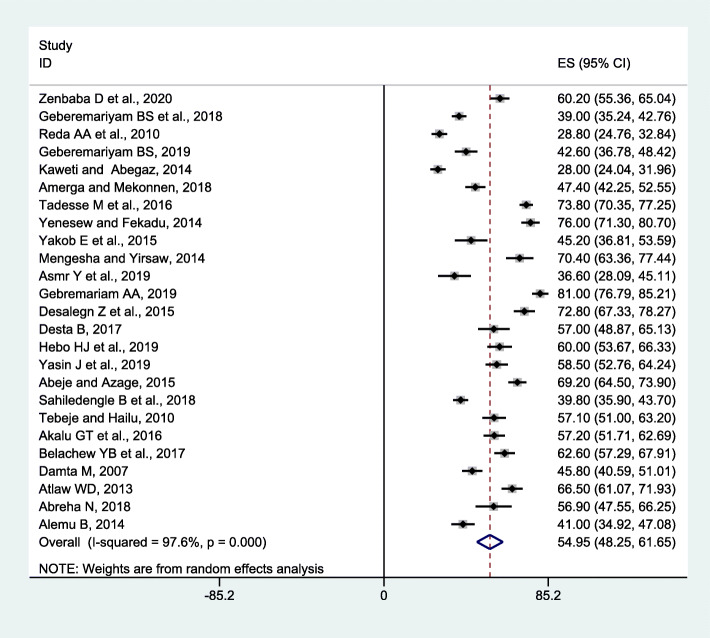
Meta-analysis, lifetime prevalence of blood and body fluid exposure among healthcare workers in Ethiopia, 2007–2020

The current meta-analysis using the random-effects model conveyed that the estimated overall pooled prevalence of 12 months BBF exposures among HCWs in Ethiopia was 44.24% (95% CI, 36.98-51.51) with a significant level of heterogeneity (*I*^2^ = 97.9%; *p* < 0.001) (Fig. 2). The lifetime pooled prevalence of BBFs using the random-effects model was 54.95% (95% CI 48.25-61.65) with a significant level of heterogeneity (*I*^2^ = 97.6%; *p* < 0.001) (Fig. 3).

### Investigation of heterogeneity and subgroup analysis

The included studies in this meta-analysis exhibited a statistically significant heterogeneity between studies (*I*^2^ = 97.9%; *p* < 0.001, and *I*^2^ = 97.6%; *p* < 0.001) for the 12-month and lifetime BBF exposure prevalence estimates, respectively. Accordingly, the random-effects model was used to adjust the observed variability. In identifying the possible source of heterogeneity, subgroup analyses were utilized based on the geographical regions, type of healthcare facilities, year of publication, and sample size. However, the level of heterogeneity between studies remained high after subgroup analysis (Table 2).

**Table 2:** Subgroup meta-analysis, blood and body fluid exposure among healthcare workers in Ethiopia, 2007–2020

Prevalence type	Variables category	Subgroup	Number of studies included	Sample size	Prevalence (95% CI)	Heterogeneity across the studies	
						*I*^2^ (%)	*P* value
Lifetime prevalence	Region	Addis Ababa	9	2473	53.00 (44.47-61.53)	94.7	< 0.001
		Oromia	6	2095	53.26 (44.03-62.49)	94.6	< 0.001
		Amhara	5	1652	66.17 (53.86-78.47)	96.9	< 0.001
		SNNP	4	1416	54.35 (28.38-80.31)	99.1	< 0.001
		Harari	1	484	28.80 (24.76-32.84)		
	Type of healthcare facility	Hospital	15	3945	55.13 (46.64-63.63)	97.0	< 0.001
		Hospital and health centers	9	3814	55.50 (43.15-67.84)	98.5	< 0.001
		Health center	1	361	47.40 (42.25-52.55)		
	Publication year	2007-2014	8	2605	51.64 (37.70-65.57)	98.3	< 0.001
		2015-2020	17	5515	56.56 (49.44-63.68)	96.8	< 0.001
	Sample size	≥ 300	13	5612	54.51 (44.13-64.89)	98.6	< 0.001
		< 300	12	2508	55.51 (48.75-62.27)	92.1	< 0.001
	Sampling technique	Probability	20	7052	54.45 (46.66-62.23)	98.0	< 0.001
		Non-probability	5	1068	57.02 (45.74-68.29)	93.1	< 0.001
	Risk of bias	Low	21	7303	53.81 (46.27-61.35)	97.9	< 0.001
		Moderate	4	817	61.27 (52.38-70.16)	85.2	< 0.001
A 12-month prevalence	Region	Addis Ababa	9	2353	39.09 (28.66-49.52)	96.8	< 0.001
		Oromia	2	624	44.19 (40.29-48.09)	0.0	0.644
		Amhara	7	2250	48.69 (35.53-61.85)	97.8	< 0.001
		Tigray	1	483	60.20 (55.83-64.57)		
		SNNP	2	1119	55.89 (36.58-75.19)	97.8	< 0.001
		Harari	2	734	31.86 (8.73-54.98)		
	Type of healthcare facility	Hospital	13	3454	45.19 (38.55-51.83)	94.5	< 0.001
		Hospital and health centers	9	3748	43.24 (28.72-57.77)	99.0	< 0.001
		Health center	1	361	40.20 (35.14-45.26)		
	Publication year	2007-2014	12	3684	47.21 (36.50-57.92)	98.0	< 0.001
		2015-2020	11	3879	41.04 (30.63-51.45)	98.0	< 0.001
	Sample size	≥ 300	11	4722	43.32 (31.52-55.12)	98.8	< 0.001
		< 300	12	2635	45.03 (37.47-52.59)	94.1	< 0.001
	Sampling technique	Probability	22	7417	44.97 (37.50-52.44)	98.0	< 0.001
		Non-probability	1	146	27.90 (20.63-35.17)		
	Risk of bias	Low	22	7417	44.97 (37.50-52.44)	98.0	< 0.001
		Moderate	1	146	27.90 (20.63-35.17)		

*SNNP* South Nation Nationalities and Peoples

The prevalence of 12 months BBFs was found to be higher in the Tigray Region 60.20% (95% CI, 55.83-64.57), and the least was reported from the Harari Region 31.86% (95% CI, 8.73-54.98). This meta-analysis also found that the lifetime prevalence of BBF exposures differed between various regions, and the highest prevalence was found in the Amhara Region, 66.17% (95% CI, 53.86-78.47), followed by SNNP Region, 54.35% (95% CI, 28.38-80.31), and finally, the least in Harari Region, 20.80% (95% CI, 24.76-32.83). Withal, the 12 months and lifetime prevalence of BBF exposures were 41.04 (95% CI, 30.63-51.45) and 56.56% (95% CI, 49.44-63.68) in studies published between 2015 and 2020, respectively (Table 2).

### Sensitivity analysis

To identify the source of heterogeneity and to explore the robustness of the findings, a leave-one-out sensitivity analysis was employed. The result of sensitivity analyses using the random-effects model revealed that no single study influenced the overall prevalence of 12 months and lifetime BBF exposures among HCWs (Additional file 4).

### The publication bias

**Fig. 4:**
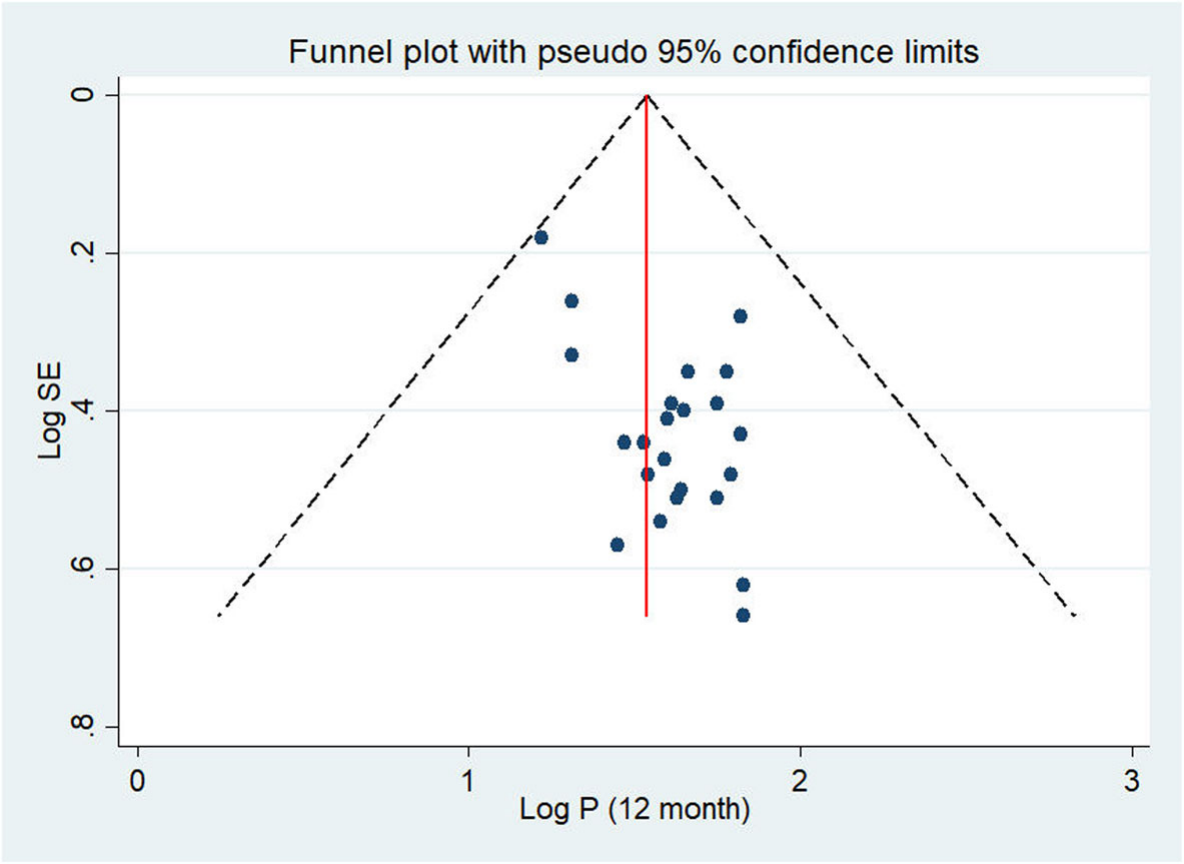
Publication bias of 12-month prevalence of BBFs exposure among HCWs in Ethiopia, 2007-2020

**Fig. 5:**
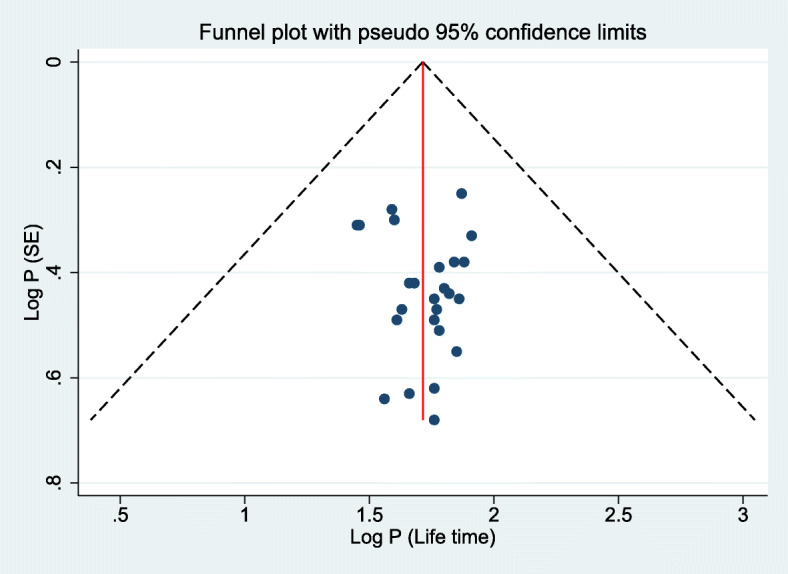
Publication bias of lifetime prevalence of BBFs exposure among HCWs in Ethiopia, 2007-2020

The presence of publication bias was evaluated using funnel plots and Egger’s tests at a significance level of less than 0.05. The findings revealed that publication bias was not significant for the studies reported in the 12-month prevalence of BBF exposures (*p* = 0.05) (Fig. 4). In the same manner, it was not statistically significant (*p* = 0.69) for the lifetime BBFs exposures, as well (Fig. 5).

### Meta-regression analysis

The results of the meta-regression analysis showed that the publication year and the sample size were not significant sources of heterogeneity. In this study, no significant relationship was identified between the 12-month prevalence of BBFs and publication year (*p* value = 0.76), and sample size (*p* value = 0.44). Similarly, there was no significant association between the lifetime prevalence of BBFs and publication year (*p* value = 0.54) and sample size (*p* value = 0.33) (Table 3).

**Table 3:** A meta-regression analysis of factors for heterogeneity of the prevalence of blood and body fluid exposure among the healthcare workers in Ethiopia, 2007-2020

Prevalence estimate	Heterogeneity source	Coefficients	Std. error	*p* value
12 months	Publication year	−0.3185998	1.026952	0.76
	Sample size	−0.0190527	0.0242865	0.44
Lifetime	Publication year	0.5911936	0.9450944	0.54
	Sample size	−0.0202265	0.0202779	0.33

### Narrative review on factors associated with BBFs exposure

As shown in Table 4, only 10 articles were reported factors associated with BBFs exposure [10, 11, 14, 19–21, 28, 29, 43, 48]. Factors related to lower odds of BBFs exposure among HCWs were type of healthcare facility [10, 28], regularly applied standard precautions [14], profession (nurse [20], midwives [48]), and receiving satisfactory infection prevention training [43] (Table 4).

**Table 4:** Studies identified factors associated with blood and body fluid exposure among healthcare workers in Ethiopia, 2007–2020

Name	Study design	Study population	Setting	Region	Results
Zenbaba D et al. [10]	CS	HCWs and C	Hospitals	Oromia	HCWs working in referral and general hospital were less likely to have blood and body fluids splash exposure as compared to primary hospitals (AOR 0.13; 95% CI 0.05-0.35) and (AOR 0.39; 95% CI 0.17-0.90), respectively. HCWs working in surgical and medical wards were more likely to have blood and body fluids splash exposure as compared to those working in OPD and laboratories (AOR 1.85; 95% CI 1.06-3.21)
Reda AA et al. [14]	CS	HCWs	Hospitals and health centers	Harari and Dire Dawa	Last 1 year incidence of needle stick injury and blood and body fluids splashing were significantly associated with each other independently (AOR 3.17; 95% CI 1.86–5.42). HCWs who had regularly applied standard precautions were less likely to had the risk of blood and body fluids splashing to the eye or mouth in the past 1 year (AOR 0.79; 95% CI 0.66–0.96).
Geberemariyam BS [11]	CS	HCWs	Hospitals	Addis Ababa	Those unmarried (single) HCWs had higher odds of blood and body fluid splash than married HCWs (AOR 1.94; 95% CI 1.18-3.41)
Amerga and Mekonnen [20]	CS	HCWs	Health centers	Addis Ababa	Profession (nurse) [AOR 0.5; 95% CI 0.26-0.98], shortage of personal protective equipment (PPE) in the past 1 year (AOR 1.86; 95% CI 1.11-3.11), not receiving training on infection prevention (AOR 7.08; 95% CI 3.57-14.03) and not using PPE (AOR 2.25; 95% CI 1.3-3.89) were factors associated with BBFs.
Tadesse M et al. [28]	CS	HCWs	Hospitals and health centers	SNNP	Profession (health officer) [AOR 1.86; 95% CI 1.02-3.38], HCWs working in gynecology ward (AOR 3.92; 95% CI 1.17-13.11), working in public health center (AOR 0.38; 95% CI 0.20-0.72), HCWs not receiving training on prevention of occupational infection (AOR 2.02; 95% CI 1.34-3.04), working in facilities that lacks safety signs (AOR 1.82; 95% CI 1.21-2.75) and lack of hand washing facilities in working department (AOR 1.82; 95% CI 1.25-2.64) were factors associated with occupational exposure to blood and body fluids.
Yenesew and Fekadu [29]	CS	HCWs	Hospitals and health centers	Amhara	Work experience (AOR 4.13; 95% CI 1.56–10.91), inconsistent use of gloves (AOR 1.98; 95% CI 1.04–3.43), and not complying with standard precautions (AOR 1.80; 95% CI 1.00–3.22) were the factors associated with occupational exposure to BBFs.
Beyera and Beyen [21]	CS	HCWs	Hospitals and health centers	Amhara	Lack of training on infection prevention (AOR 4.49; 95% CI 2.27, 8.89), long working hours per week (AOR 9.8; 95% CI 5.13, 18.74), 5–10 years work experience (AOR 2.81; 95% CI 1.15, 6.86), absence of work guidelines (AOR 2.06; 95% CI 1.03, 4.1), and dissatisfaction with current job (AOR 6.62; 95% CI 3.53, 12.43) were factors independently associated with BBFs exposure.
Yasin J et al. [19]	CS	HCWs	Hospital	Amhara	Training on infection prevention (AOR 2.17; 95% CI 1.25, 3.7), not wearing eye goggle (AOR 2.29; 95% CI 1.14, 4.6), and having HBV vaccination (AOR 1.82; 95% CI 1.08, 3.03) were factors associated with occupational exposure to BBFs splash.
Yimechew Z et al .[43]	CS	HCWs and C	Hospital	Amhara	Profession (interns) [AOR 9.4; 95% CI 1.8-49.9], 2–4 years work experience (AOR 3.2; 95% CI 1.4-7.5), and satisfactory infection prevention training (AOR 0.5; 95% CI 0.3-0.9) were factors associated with occupational exposure to BBFs splash.
Girmaye E et al. [48]	CS	HCWs and C	Hospital	Addis Ababa	Profession (midwives) [AOR 0.02; 95% CI 0.01-0.41], working for 40 and more hours (AOR 5.85; 95% CI 1.29-26.6), and vaccinated against HBV (AOR 3.57; 95% CI 1.07-11.80) were factors associated with blood and body fluid splash.

*HCWs* healthcare workers, *C* cleaners/waste handlers, *CS* cross-sectional study design, *AOR* adjusted odds ratio

## Discussion

Each year, hundreds of thousands of HCWs, including waste handlers, face the risk of blood-borne diseases due to occupational BBF exposures [3, 8, 9, 50].. In Ethiopia, despite the recognition on the importance of HBV, HCV, HIV, and other diseases transmitted through BBFs by the Federal Ministry of Health (FMoH), currently, there is dearth of systematic reviews and meta-analyses that estimated the prevalence of BBFs exposure among HCWs. In this reckon, this study was the first systematic review and meta-analysis that aimed to estimate lifetime and a 12-month prevalence on occupational exposure to BBFs among Ethiopian HCWs. This review involved the results of 36 articles, which investigated the prevalence of BBF exposures, and a high burden on occupational exposures to BBFs among HCWs in Ethiopia was evidently identified.

The estimated pooled 12 months and lifetime prevalence on BBF exposures among HCWs in Ethiopia were 44.2% and 54.9%, respectively. Forbye, the 12-month BBFs prevalence in the primary studies ranged from 16.5 [12] to 67.5% [23]. In parallel, the lifetime prevalence ranged from 28.8 [14] and 81.0% [33]. This 12-month pooled prevalence estimate was almost comparable from the pooled estimate from East Africa (47.3%) [9], Côte d’Ivoire, Mali, and Senegal (45.7%) [51], and a study by Bi P et al. from Australia, revealed that 42% of HCWs had body fluid exposures in a year on their study [52]. However, it was lower than the studies conducted in Turkey (57%) [53] and Nigeria 67.5% [54]. These differences might be subjected to the variances in the socio-demographic, cultural characteristics of study participants, and study health facility setup variations.

This study explicated a higher prevalence of lifetime BBF exposures (54.9%); however, it was subservient than the reviews from the 65 studies in 21 African countries (65.7%) [9]. The foremost reason for this variation may be due to study setting dissimilarities. This finding is also inconsistent with a study in Iran which reported the prevalence of exposures at 46.47% [55]. The variance could be due to the discrepancies in the study participants (service personnel, paramedics, and nursing students were included) [55], types of included studies (historical cohort study were included in Iranian review) [55], the type of healthcare facilities (in the present review we included HCWs from primary healthcare units), and socio-demographic factors [9]. Generally, the lifetime BBF exposure rate in this study (54.9%) seems to be very low when we compared with the annual BBF exposure rate 44%. One possible explanation of why the lifetime BBF exposure rate is relatively lower in this study was due to recall bias, which is a potential limitation in self-reported studies. The other possible reason is that BBFs exposure was underreported in some of the included studies, which is likely. As all included studies were cross-sectional studies we detected relatively lower lifetime BBFs exposure.

In this review, the researchers identified a variation in the HCWs’ exposure to BBFs across the Ethiopian regions. The lifetime (66.17% in Amhara Region) and 12 months (60.20% in Tigray Region and 48.69% in Amhara Region) occupational exposure to BBFs were consistently more frequent in Northern Ethiopia, and less in Harari Region (lifetime prevalence of 28.80% and 12-month prevalence of 31.86%). The probable rationale for these regional variations may be due to the number of studies included; type of healthcare facilities; and geographical and demographical differences. The other possible vindication for these disparities may be partially explained by the polarities in the levels of standard precaution practices among the HCWs in the various regions. As one study reported, 80.8% of the HCWs regularly follow standard precautions in Eastern Ethiopia, including the Harari Region [14].

A laudative prevalence of BBF exposures among HCWs working exclusively in hospitals than those in the health centers (primary healthcare units) was also found. Almost half of the HCWs working in hospitals of Ethiopia had at least one BBFs exposure in their lifetime and in the last 12 months. The finding was predictable because these HCWs had higher workloads and they performed further medical procedures, which may have exposed them to occupational BBFs compared to those in the health centers. Therewithal, the high prevalence of BBF exposures among HCWs working in the hospitals had significant implications because most of the blood-borne viruses, such as HCV, HBV, and HIV, may haply spread through BBFs exposures, therefore, enhancing HCWs’ compliance toward standard precautionary measures is deemed necessary.

Up to date, no specific reporting guidelines have been available in the country solely on BBFs exposures. To overcome the high prevalence of mucocutaneous injury among healthcare workers in Ethiopia, the Ministry of Health should take the lead in the development of reporting guideline and in settings of standards in order to close monitoring of BBFs exposure in the country. As this review includes cross-sectional study, the limitations that come with this type of design need to be taken into consideration when interpreting the findings; it is recommended that future prospective research investigate the incidence of occupational exposure to blood and body fluids, the preventive measures, and the circumstances in which it occurs is required. Further, it will be important for implementation of preventative policies and interventions based on current knowledge to minimize the high burden of BBFs exposure in Ethiopia.

### Limitations

This review article had a few adversities due to its limitations. One of which was the cross-sectional design nature of the included studies and all were based on self-reported data while estimating the prevalence of occupational BBFs exposures. Additionally, social desirability and recall biases were likely present. Since the study was conducted in Ethiopia, included healthcare facilities and the generalization of the study findings were limited to these similar contexts. Further, there was no study obtained from some Ethiopian regions, such as Afar Regional State and Benshangul-Gumuz Regional State and this might probably affect the generalizability of the present findings at a national level. Furthermore, there remains a pressing need for high-quality data on occupational BBFs exposure to identify preventive measures. Finally, attempts were made to include all the published articles on factors associated with BBFs exposure, but it is likely that some important risk factor findings have not been included mainly because of the type of the search strategy and type of study design adopted in this review. We did not also analyzed the effects of experience years of medical staff and the adoption of personal protective equipment including goggles and face shields in the reduction of mucosal exposure incidents.

## Conclusions

This review exhibited a higher percentage of occupational exposures to BBFs among HCWs in Ethiopia. The available evidences suggest that more than two-in-five and one-half of healthcare workers in Ethiopia were exposed to BBFs annually and in their lifetime, respectively. Therefore, efforts should be implemented to reduce the high burden of occupational blood and body fluid exposures through effective implementation of standard precaution measures along with aggressive occupational health and safety activities.

## Supplementary information

**Additional file 1:.** PRISMA checklist.

**Additional file 2:.** Examples of search strategy.

**Additional file 3:.** The risk-of-bias assessment results for included studies.

**Additional file 4:.** Sensitivity analysis for included studies of BBFs.

## Abbreviations

AOR: Adjusted odds ratio
BBFs: Blood and body fluids
HCWs: Healthcare workers
CI: Confidence interval
IPPS: Infection Prevention and Patient Safety
PRISMA: Preferred Reporting Items for Systematic Reviews and Meta-Analyses
WHO: World Health Organization

## References

[1] Westermann C, Peters C, Lisiak B, Lamberti M, Nienhaus A (2015). The prevalence of hepatitis C among healthcare workers: a systematic review and meta-analysis. Occup Environ Med..

[2] Deuffic-Burban S, Delarocque-Astagneau E, Abiteboul D, Bouvet E, Yazdanpanah Y (2011). Blood-borne viruses in health care workers: prevention and management. J Clinical Virology..

[3] World Health Organization [Internet]. Health Care Worker Safety 2016. [cited 2020 March 23]. Available from: http://www.who.int/injection_safety/toolbox/en/AM_HCW_Safety_EN.pdf.

[4] Tavoschi L, Mason L, Petriti U, Bunge E, Veldhuijzen I, Duffell E (2019). Hepatitis B and C among healthcare workers and patient groups at increased risk of iatrogenic transmission in the European Union/European Economic Area. Journal of Hospital Infection..

[5] Janine J, Jane P. Avoiding blood and body fluid exposures, Nursing 2002; 2002; 32(8): 68. 10.1097/00152193-200208000-00054 12360918

[6] Tarantola A, Abiteboul D, Rachline A (2006). Infection risks following accidental exposure to blood or body fluids in health care workers: a review of pathogens transmitted in published cases. Am J Infect Control..

[7] Motaarefi H, Mahmoudi H, Mohammadi E, Hasanpour-dehkordi A (2016). Factors Associated with Needlestick Injuries in Health Care Occupations: A Systematic Review. Journal of Clinical and Diagnostic Research..

[8] Mossburg S, Agore A, Nkimbeng M, Commodore-Mensah Y (2019). Occupational Hazards among Healthcare Workers in Africa: A Systematic Review. Annals of Global Health..

[9] Auta A, Adewuyi EO, Tor-Anyiin A, Aziz D, Ogbole E, Ogbonna BO, Adeloye D (2017). Health-care workers’ occupational exposures to body fluids in 21 countries in Africa: systematic review and meta-analysis. Bull. World Health Organ..

[10] Zenbaba D, Bogale D, Sahiledengle B, Woldeyohannes D, Tekalegn Y. Prevalence and factors associated with needle-stick injuries and splash with blood and body fluids among healthcare workers in hospitals of Bale Zone, Southeast Ethiopia. Ethiop Med J. 2020;58(01).

[11] Gebremariyam BS. Determinants of occupational exposure to blood and body fluids, healthcare workers’ risk perceptions and standard precautionary practices: A hospital-based study in Addis Ababa, Ethiopia. Ethiop J Health Dev. 2019;33(1).

[12] Sahiledengle B, Gebersilassie A, Desta H, Tadesse G (2018). Infection prevention practices and associated factors among healthcare workers in governmental healthcare facilities in Addis Ababa. Ethiopia. Ethiop J Health Sci..

[13] Geberemariyamet BS, Donka G, Wordofa B (2018). Assessment of knowledge and practices of healthcare workers towards infection prevention and associated factors in healthcare facilities of West Arsi District. Southeast Ethiopia: a facility-based cross-sectional study. Arc Public Health..

[14] Reda AA, Fisseha S, Mengistie B, Vandeweerd JM. Standard precautions: occupational exposure and behavior of health care workers in Ethiopia. PLoS One. 2010;5(12). 10.1371/journal.pone.0014420 PMC3009714 21203449

[15] Zenbaba D, Sahiledengle B, Bogale D. Practices of Healthcare Workers regarding Infection Prevention in Bale Zone Hospitals. Southeast Ethiopia. Adv Public Health. 2020;2020.

[16] Gebresilassie A, Kumei A, Yemane D (2014). Standard precautions practice among health care workers in public health facilities of Mekelle special zone, Northern Ethiopia. J Community Med Health Educ..

[17] Belachew YB, Lema TB, Germossa GN, Adinew YM (2017). Blood/body fluid exposure and needle stick/sharp injury among nurses working in public hospitals. Southwest Ethiopia. Front Public Health..

[18] Alemayehu T, Worku A, Assefa N (2016). Medical waste collectors in eastern Ethiopia are exposed to high sharp injury and blood and body fluids contamination. Prev Inf Cntrl..

[19] Yasin J, Fisseha R, Mekonnen F, Yirdaw K (2019). Occupational exposure to blood and body fluids and associated factors among health care workers at the University of Gondar Hospital. Northwest Ethiopia. Environ Health Prev Med..

[20] Amerga EW, Mekonnen TG (2018). Occupational Exposure to Blood and Body Fluids among Health Care Workers in Arada Sub-city Health Centers of Addis Ababa. Ethiopia. Occup Med Health Aff..

[21] Beyera GK, Beyen TK (2014). Epidemiology of exposure to HIV/AIDS risky conditions in healthcare settings: the case of health facilities in Gondar City. North West Ethiopia. BMC Public Health..

[22] Kaweti G, Abegaz T (2015). Prevalence of percutaneous injuries and associated factors among health care workers in Hawassa referral and adare District hospitals, Hawassa, Ethiopia, January 2014. BMC Public Health..

[23] Shiferaw Y, Abebe T, Mihret A (2012). Sharps injuries and exposure to blood and bloodstained body fluids involving medical waste handlers. Waste Manag Res..

[24] Yazie TD, Chufa KA, Tebeje MG (2019). Prevalence of needlestick injury among healthcare workers in Ethiopia: a systematic review and meta-analysis. Environ Health Prev Med..

[25] Moher D, Liberati A, Tetzlaff J, Altman DG. Group TP, Oxman A, Cook D, Guyatt G, Swingler G, Volmink J, Ioannidis J, Young C, Horton R, et al. Preferred Reporting Items for Systematic Reviews and Meta-Analyses: The PRISMA Statement. PLoS Med. 2009;6:e1000097. 10.1371/journal.pmed.1000097 PMC2707599 19621072

[26] The Joanna Briggs Institute. Critical appraisal tools for use in JBI systematic reviews checklist for prevalence studies: The University of Adelaide. [cited 2020 February 10 ]. Available from: https://joannabriggs.org/sites/default/files/2019-05/JBI_ Critical_Appraisal Checklist_for_Prevalence_Studies2017_0.pdf.

[27] Amare Z, Sheng W, Hussien A (2018). Dawit. Assessment of knowledge, attribution and practice related to NSIS and blood exposure among health care workers in the armed forces referral and teaching hospital, Addis Ababa, Ethiopia. Int J Adv Res..

[28] Tadesse M, Meskele M, Tadesse A (2016). Occupational exposure to blood and body fluids among health care workers in Wolaita Zone. Southern Ethiopia. Developing Country Studies..

[29] Yenesew MA, Fekadu GA (2014). Occupational exposure to blood and body fluids among health care professionals in Bahir Dar town. Northwest Ethiopia. Saf Health Work..

[30] Yakob E, Lamaro T, Henok A (2015). Knowledge, attitude and practice towards infection control measures among Mizan-Aman general hospital workers, South West Ethiopia. J Community Med Health Educ..

[31] Beyene H, Yirsaw BD (2014). Occupational risk factors associated with needle-stick injury among healthcare workers in Hawassa City. Southern Ethiopia. Occup Med Health Aff..

[32] Asmr Y, Beza L, Engida H, Bekelcho T, Tsegaye N, Aschale Y. Assessment of knowledge and practices of standard precaution against blood borne pathogens among doctors and nurses at adult emergency room in Addis Ababa. Ethiopia. Emerg Med Int. 2019;2019. 10.1155/2019/2926415 PMC6507162 31179129

[33] Gebremariam AA, Tsegaye AT, Shiferaw YF, Reta MM, Getaneh A. Seroprevalence of hepatitis B virus and associated factors among health professionals in University of Gondar Hospital. Northwest Ethiopia. Adv Prev Med. 2019;2019. 10.1155/2019/7136763 PMC6421023 30941224

[34] Desalegn Z, Gebreselassie S, Asemamaw Y (2015). Epidemiology of needle stick-sharp injuries (NSSIs) and potential high risk exposures among health professionals in Ethiopia: neglected public health concern. Am J Health Res..

[35] Jemaneh L. Assessment of knowledge, attitude and practice among health care workers regarding needle stick and sharp object injuries in Army force Referral and teaching hospital, Addis Ababa, Ethiopia (Doctoral dissertation, Addis Ababa University).

[36] Desta B. Assessment of Knowledge, Attitude and Practice of Nurses Working in Adult and Pediatric ICU and Emergency Department Towards Standard Precausions at Tikur Anbesa Specialized Hospital from December 2016 To June 2017 (Doctoral dissertation, Addis Ababa University).

[37] Aynalem Tesfay F, Dejenie HT. Assessment of prevalence and determinants of occupational exposure to HIV infection among healthcare workers in selected health institutions in Debre Berhan town, North Shoa Zone, Amhara Region, Ethiopia, 2014. AIDS Res Treat. 2014;2014. 10.1155/2014/731848 PMC4247935 25478213

[38] Worku W. Hospital Acquired Infections and Infection Prevention Practice in Teaching Hospitals in the Amhara Regional State, Ethiopia (Doctoral dissertation, Addis Ababa University).

[39] Hebo HJ, Gemeda DH, Abdusemed KA. Hepatitis B and C viral infection: prevalence, knowledge, attitude, practice, and occupational exposure among healthcare workers of Jimma University Medical Center, southwest Ethiopia. Sci World J. 2019;2019. 10.1155/2019/9482607 PMC6377947 30853866

[40] Abeje G, Azage M (2015). Hepatitis B vaccine knowledge and vaccination status among health care workers of Bahir Dar City Administration, Northwest Ethiopia: a cross sectional study. BMC Infect Dis.

[41] Tebeje B, Hailu C. Assessment of HIV post-exposure prophylaxis use among health workers of governmental health institutions in Jimma Zone, Oromiya Region, Southwest Ethiopia. Ethiop J Health Sci. 2010;20(1). 10.4314/ejhs.v20i1.69429 PMC3275901 22434961

[42] Akalu GT, Woldemariam AT, Shewaye AB, Geleta DA, Demise AH, Debele MT (2016). Burden of hepatitis-B infections and risk factors among healthcare workers in resource limited setting, Addis Ababa. Ethiopia. EC Microbiol..

[43] Yimechew Z, Tiruneh G, Ejigu T. Occupational exposures to blood and body fluids (BBFS) among health care workers and medical students in University of Gondar Hospital, Northwest of Ethiopia. Glob J Med Res. 2013.

[44] Damte M (2006). Assessment of the Knowledge, Attitude and Practice of Health Care Workers on Universal Precaution in North Wollo Zone.

[45] Atlaw WD. Patterns of occupational exposure to patients' body fluids among health care workers in Tikuranbesa University Hospital, Addis Ababa, Ethiopia (Doctoral dissertation).

[46] Gebreselassie FT. Investigating the compliance with universal precautions among health care providers in Tikur Anbessa Central Referral Hospital, Addis Ababa, Ethiopia (Doctoral dissertation, University of Western Cape).

[47] Abreha N. Assessment of knowledge and practice towards infection prevention and associated factors among nurses working in adult and pediatric emergency in Tikur Anbessa specialized hospital, Addis Ababa, Ethiopia (Doctoral dissertation, Addis Ababa Universty).

[48] Girmaye E, Belema D, Mamo K, Daba G. Assesment of Percutaneous Exposure Incidents and Associated Factors among Health Care Personnel in Gandhi Memorial Hospital. Addis Ababa. J Health Med Nurs. 2018;52.

[49] Alemu B. Awareness of Hiv Post-Exposure Prophylaxis Among Health Care Personnel in Asella Teaching Hospital, Asella Town, South-East Ethiopia (Doctoral dissertation, Addis Ababa University).

[50] Chalya PL, Seni J, Mushi MF, Mirambo MM, Jaka H, Rambau PF, Kapesa A, Ngallaba SE, Massinde AN, Kalluvya SE. Needle-stick injuries and splash exposures among health-care workers at a tertiary care hospital in north-western Tanzania. Tanzan J Health Res. 2015;17(2).

[51] Tarantola A, Koumare A, Rachline A, Sow PS, Diallo MB, Doumbia S, Aka C, Ehui E, Brücker G, Bouvet E (2005). Groupe d'Etude des Risques d'Exposition des Soignants aux agents infectieux. A descriptive, retrospective study of 567 accidental blood exposures in healthcare workers in three West African countries. J Hosp Infect..

[52] Bi P, Tully PJ, Boss K, Hiller JE (2008). Sharps injury and body fluid exposure among health care workers in an Australian tertiary hospital. Asia Pac J Public Health..

[53] Irmak Z, Baybuga MS (2011). Needlestick and sharps injuries among Turkish nursing students: A cross‐sectional study. Int J Nurs Pract..

[54] Nwankwo TO, Aniebue UU. Percutaneous injuries and accidental blood exposure in surgical residents: Awareness and us of prophylaxis in relation to HIV. Niger J Clin Pract. 2011;14(1). 10.4103/1119-3077.79237 21493989

[55] Fereidouni Z, Kameli Morandini M, Dehghan A, Jamshidi N, Najafi KM (2018). The prevalence of needlestick injuries and exposure to blood and body fluids among Iranian healthcare workers: a systematic review. Int J Med Rev..

