# Supplementary material for: Occupational exposures to blood and body fluids among healthcare workers in Ethiopia: a systematic review and meta-analysis
Source: Environ Health Prev Med. 2020 Oct 3;25:58. doi: 10.1186/s12199-020-00897-y (PMC7533038; doi:10.1186/s12199-020-00897-y)
Supplement: Supplementary file 2 — Additional file 2:. Examples of search strategy. [file 12199_2020_897_MOESM2_ESM.docx]

**PubMed**

1. Occupational Exposure [MeSH Terms] OR Occupation* exposure [All Fields] OR Accidents [MeSH Terms] OR Accident [All Fields] OR Accident* exposure [All Fields] OR Occupational Disease [MeSH Terms] OR Occupation* disease [All Fields] OR Accidental blood disease* [All Fields] OR Accidental occupational exposure [All Fields] OR Occupational hazard* [All Fields] OR Occupational transmission [All Fields] OR Cross infection [MeSH Terms]
2. Blood [MeSH Terms] OR Body Fluids [MeSH Terms] OR Body fluid* [All Fields] OR blood spill* [All Fields] OR Blood-Borne Pathogens [MeSH Terms] OR Blood borne pathogen* [All Fields] OR Blood-borne infection* [All Fields] OR percutaneous injur* [All Fields] OR mucus membrane exposure* [All Fields] OR nonintact skin exposure* [All Fields] OR HIV [MeSH Term] OR Hepatitis B [MeSH Term] OR Hepatitis C [MeSH Term] OR Universal Precautions [MeSH Terms] OR universal precaution [All Fields].
3. Health personnel [MeSH Term] OR Health care worker* [All Fields] OR Health worker* [All Fields] OR Health staff [All Fields] OR Medical personnel [All Fields] OR Health Occupations [MeSH Terms] OR Nurses [MeSH Terms] OR Midwive* [All Fields] OR Physicians [MeSH Terms] OR Surgeons [MeSH Terms] OR Health officer* [All Fields]
4. Ethiopia [MeSH Terms]
5. 1 OR 2
6. 5 AND 3
7. 6 AND 4

Full searching

((((((((((((((((Occupational Exposure[MeSH Terms]) OR "Occupation* exposure") OR Accidents[MeSH Terms]) OR "Accident* exposure") OR Occupational Disease[MeSH Terms]) OR "Occupation* disease") OR "Accidental blood disease*") OR "Accidental occupational exposure") OR "Occupational hazard*") OR "Occupational transmission") OR Cross infection[MeSH Terms])) OR (((((((((((((((Blood[MeSH Terms]) OR Body Fluids[MeSH Terms]) OR "Body fluid*") OR "blood spill*") OR Blood-Borne Pathogens[MeSH Terms]) OR "Blood borne pathogen*") OR "Blood-borne infection*") OR "percutaneous injur*") OR "mucus membrane exposure*") OR "nonintact skin exposure*") OR HIV[MeSH Terms]) OR Hepatitis B[MeSH Terms]) OR Hepatitis C[MeSH Terms]) OR Universal Precautions[MeSH Terms]) OR "universal precaution"))) AND (((((((((((Health personnel[MeSH Terms]) OR "Health care worker*") OR "Health worker*") OR "Health staff") OR "Medical personnel") OR "Health Occupations"[MeSH Terms]) OR Nurses[MeSH Terms]) OR "Midwive*") OR Physicians[MeSH Terms]) OR Surgeons[MeSH Terms]) OR "Health officer*"))) AND Ethiopia[MeSH Terms].

Overall History

(((((((((((("occupational exposure"[MeSH Terms] OR "Occupation* exposure"[All Fields]) OR "accidents"[MeSH Terms]) OR "Accident* exposure"[All Fields]) OR "occupational diseases"[MeSH Terms]) OR "Occupation* disease"[All Fields]) OR (("accidents"[MeSH Terms] OR "accidents"[All Fields] OR "accidental"[All Fields]) AND ("hematologic diseases"[MeSH Terms] OR ("hematologic"[All Fields] AND "diseases"[All Fields]) OR "hematologic diseases"[All Fields] OR ("blood"[All Fields] AND "disease"[All Fields]) OR "blood disease"[All Fields]))) OR "Accidental occupational exposure"[All Fields]) OR "Occupational hazard*"[All Fields]) OR "Occupational transmission"[All Fields]) OR "cross infection"[MeSH Terms]) OR (((((((((((((("blood"[MeSH Terms] OR "body fluids"[MeSH Terms]) OR "Body fluid*"[All Fields]) OR (("blood"[Subheading] OR "blood"[All Fields] OR "blood"[MeSH Terms]) AND (spill[All Fields] OR spill'[All Fields] OR spill's[All Fields] OR spilla[All Fields] OR spilla1[All Fields] OR spillage[All Fields] OR spillage'[All Fields] OR spillaged[All Fields] OR spillages[All Fields] OR spillages'[All Fields] OR spillai[All Fields] OR spillaine[All Fields] OR spillan[All Fields] OR spillance[All Fields] OR spillane[All Fields] OR spillantini[All Fields] OR spillar[All Fields] OR spillard[All Fields] OR spillare[All Fields] OR spillars[All Fields] OR spillay[All Fields] OR spillback[All Fields] OR spillback'[All Fields] OR spillbacks[All Fields] OR spillberg[All Fields] OR spillberger's[All Fields] OR spillbrunnssystemet[All Fields] OR spillcanvas29[All Fields] OR spillcontingency[All Fields] OR spilldown[All Fields] OR spille[All Fields] OR spilleavhengighet[All Fields] OR spillebeen[All Fields] OR spilleboudt[All Fields] OR spillebout[All Fields] OR spillecke[All Fields] OR spilled[All Fields] OR spilleers[All Fields] OR spillekom[All Fields] OR spillelidenskab[All Fields] OR spillemaecker[All Fields] OR spillemaeckers[All Fields] OR spillemaeker[All Fields] OR spillemani[All Fields] OR spillemose[All Fields] OR spillenaar[All Fields] OR spillenberger[All Fields] OR spillenbergerovcov[All Fields] OR spillenger[All Fields] OR spiller[All Fields] OR spiller's[All Fields] OR spillera[All Fields] OR spillerb[All Fields] OR spilleregler[All Fields] OR spillerestefanny[All Fields] OR spillerk[All Fields] OR spillerl[All Fields] OR spillerova[All Fields] OR spillers[All Fields] OR spillert[All Fields] OR spillerum[All Fields] OR spilles[All Fields] OR spillesyge[All Fields] OR spillet[All Fields] OR spillets[All Fields] OR spillett[All Fields] OR spillgellberg[All Fields] OR spillheide[All Fields] OR spilli[All Fields] OR spilliaert[All Fields] OR spillidan[All Fields] OR spillier[All Fields] OR spilling[All Fields] OR spilling'[All Fields] OR spillinger[All Fields] OR spillings[All Fields] OR spilliotis[All Fields] OR spillith[All Fields] OR spillius[All Fields] OR spillman[All Fields] OR spillman's[All Fields] OR spillmann[All Fields] OR spillmann's[All Fields] OR spillmannj[All Fields] OR spillner[All Fields] OR spillo[All Fields] OR spillone[All Fields] OR spilloproject[All Fields] OR spillotis[All Fields] OR spillout[All Fields] OR spillover[All Fields] OR spillover'[All Fields] OR spillovers[All Fields] OR spillovers'[All Fields] OR spillovet[All Fields] OR spillp[All Fields] OR spillproof[All Fields] OR spills[All Fields] OR spills'[All Fields] OR spillsbury[All Fields] OR spillson[All Fields] OR spillsurveillance[All Fields] OR spilltrays[All Fields] OR spillum[All Fields] OR spillunder[All Fields] OR spillunders[All Fields] OR spillus[All Fields] OR spillway[All Fields] OR spillways[All Fields] OR spilly[All Fields]))) OR "blood-borne pathogens"[MeSH Terms]) OR "Blood borne pathogen*"[All Fields]) OR "Blood-borne infection*"[All Fields]) OR (percutaneous[All Fields] AND (injur[All Fields] OR injura[All Fields] OR injurable[All Fields] OR injural[All Fields] OR injurance[All Fields] OR injurant[All Fields] OR injurants[All Fields] OR injuray[All Fields] OR injurdata[All Fields] OR injure[All Fields] OR injure'[All Fields] OR injured[All Fields] OR injured'[All Fields] OR injured's[All Fields] OR injureda[All Fields] OR injuredbrachygobius[All Fields] OR injuredness[All Fields] OR injuredpatients[All Fields] OR injureds[All Fields] OR injureis[All Fields] OR injurer[All Fields] OR injurer's[All Fields] OR injureries[All Fields] OR injurers[All Fields] OR injurers'[All Fields] OR injures[All Fields] OR injuresat[All Fields] OR injurey[All Fields] OR injuri[All Fields] OR injuria[All Fields] OR injuriadas[All Fields] OR injuriados[All Fields] OR injuriante[All Fields] OR injuriarum[All Fields] OR injurias[All Fields] OR injuribility[All Fields] OR injurics[All Fields] OR injuridest[All Fields] OR injurie[All Fields] OR injuried[All Fields] OR injurier[All Fields] OR injuriers[All Fields] OR injuries[All Fields] OR injuries'[All Fields] OR injuries's[All Fields] OR injuries1[All Fields] OR injuries15[All Fields] OR injuriesand[All Fields] OR injuriesat[All Fields] OR injuriescommittee[All Fields] OR injuriesconflicts[All Fields] OR injuriesdagger[All Fields] OR injuriesfaculty[All Fields] OR injurieshave[All Fields] OR injuriesin[All Fields] OR injuriesof[All Fields] OR injuriesrelated[All Fields] OR injuriesthat[All Fields] OR injurieswere[All Fields] OR injuriies[All Fields] OR injurin[All Fields] OR injurin'[All Fields] OR injuring[All Fields] OR injuring'[All Fields] OR injurins[All Fields] OR injurins'[All Fields] OR injuriologists[All Fields] OR injurious[All Fields] OR injurious'[All Fields] OR injuriously[All Fields] OR injuriousness[All Fields] OR injurires[All Fields] OR injuris[All Fields] OR injurites[All Fields] OR injurity[All Fields] OR injuriy[All Fields] OR injurles[All Fields] OR injurof[All Fields] OR injurous[All Fields] OR injurry[All Fields] OR injurt[All Fields] OR injurty[All Fields] OR injurues[All Fields] OR injury[All Fields] OR injury'[All Fields] OR injury''[All Fields] OR injury'is[All Fields] OR injury's[All Fields] OR injury,[All Fields] OR injury1[All Fields] OR injury13[All Fields] OR injury360[All Fields] OR injury9[All Fields] OR injury9,10[All Fields] OR injury`s[All Fields] OR injurya[All Fields] OR injuryabsolute[All Fields] OR injuryan[All Fields] OR injuryand[All Fields] OR injuryapache[All Fields] OR injuryassociated[All Fields] OR injuryassociation[All Fields] OR injuryautologous[All Fields] OR injurybeijing[All Fields] OR injurycaremedical[All Fields] OR injuryccr2[All Fields] OR injurycin[All Fields] OR injuryclinodactyly[All Fields] OR injurycollaborative[All Fields] OR injurycontrol[All Fields] OR injurycta[All Fields] OR injurydagger[All Fields] OR injuryed[All Fields] OR injuryeffect[All Fields] OR injuryfor[All Fields] OR injuryfree[All Fields] OR injuryfuture[All Fields] OR injuryhemerocallis[All Fields] OR injuryhsp[All Fields] OR injuryies[All Fields] OR injuryimpact[All Fields] OR injuryin[All Fields] OR injuryinduced[All Fields] OR injurying[All Fields] OR injuryintervention[All Fields] OR injuryintrathecal[All Fields] OR injuryissued[All Fields] OR injuryjournal[All Fields] OR injuryleads[All Fields] OR injuryliri[All Fields] OR injurym[All Fields] OR injurymanagementpartners[All Fields] OR injuryncaa[All Fields] OR injuryof[All Fields] OR injuryprediction[All Fields] OR injuryprev[All Fields] OR injuryprevention[All Fields] OR injuryproducing[All Fields] OR injuryrats[All Fields] OR injuryreperfusion[All Fields] OR injurys[All Fields] OR injurysci[All Fields] OR injuryscore[All Fields] OR injuryserum[All Fields] OR injurysimulating[All Fields] OR injurystatistical[All Fields] OR injurystir[All Fields] OR injuryt'sharps[All Fields] OR injurytdermatologic[All Fields] OR injuryte[All Fields] OR injuryteam[All Fields] OR injurythan[All Fields] OR injurythe[All Fields] OR injurythymoquinone[All Fields] OR injuryto[All Fields] OR injurytotreatment[All Fields] OR injurytrack[All Fields] OR injurytrends[All Fields] OR injurytriggered[All Fields] OR injurytsci[All Fields] OR injuryuch[All Fields] OR injuryupdate[All Fields] OR injuryvitamin[All Fields] OR injurywas[All Fields] OR injurywasassessedby[All Fields] OR injurywhen[All Fields]))) OR (("mucus"[MeSH Terms] OR "mucus"[All Fields]) AND ("membranes"[MeSH Terms] OR "membranes"[All Fields] OR "membrane"[All Fields]) AND (exposure[All Fields] OR exposure'[All Fields] OR exposure''[All Fields] OR exposure's[All Fields] OR exposure,[All Fields] OR exposure1[All Fields] OR exposure6[All Fields] OR exposure96to[All Fields] OR exposurea[All Fields] OR exposureabsorptionexcretion[All Fields] OR exposureand[All Fields] OR exposureannoyance[All Fields] OR exposureas[All Fields] OR exposureassessment[All Fields] OR exposurebehandeling[All Fields] OR exposurecontrol[All Fields] OR exposured[All Fields] OR exposuredagger[All Fields] OR exposuredisease[All Fields] OR exposuredoses[All Fields] OR exposurefor[All Fields] OR exposurefrom[All Fields] OR exposurei[All Fields] OR exposureinduced[All Fields] OR exposureing[All Fields] OR exposureis[All Fields] OR exposurelhth[All Fields] OR exposurement[All Fields] OR exposuremeter[All Fields] OR exposureof[All Fields] OR exposureon[All Fields] OR exposurepathways[All Fields] OR exposurequality[All Fields] OR exposurerelated[All Fields] OR exposurereport[All Fields] OR exposureresponse[All Fields] OR exposureresulted[All Fields] OR exposurereveals[All Fields] OR exposures[All Fields] OR exposures'[All Fields] OR exposuresmultiple[All Fields] OR exposuresto[All Fields] OR exposuretcdd[All Fields] OR exposurethe[All Fields] OR exposureto[All Fields] OR exposuretob[All Fields] OR exposuretotwo[All Fields] OR exposureuptake[All Fields] OR exposureuva[All Fields] OR exposurewindow[All Fields] OR exposureworldwide[All Fields] OR exposurexage[All Fields] OR exposurexbody[All Fields] OR exposurexconcentration[All Fields] OR exposurexrace[All Fields] OR exposurextime[All Fields]))) OR "nonintact skin exposure*"[All Fields]) OR "hiv"[MeSH Terms]) OR "hepatitis b"[MeSH Terms]) OR ("hepatitis c"[MeSH Terms] OR "hepacivirus"[MeSH Terms])) OR "universal precautions"[MeSH Terms]) OR "universal precaution"[All Fields])) AND (((((((((("health personnel"[MeSH Terms] OR "Health care worker*"[All Fields]) OR "Health worker*"[All Fields]) OR "Health staff"[All Fields]) OR "Medical personnel"[All Fields]) OR "Health Occupations"[MeSH Terms]) OR "nurses"[MeSH Terms]) OR "Midwive*"[All Fields]) OR "physicians"[MeSH Terms]) OR "surgeons"[MeSH Terms]) OR "Health officer*"[All Fields])) AND "ethiopia"[MeSH Terms] AND ("0001/01/01"[PDAT] : "2020/01/31"[PDAT])

**Hinari**

1. “Blood” OR “Body Fluid”
2. “Exposure” AND “Occupational”
3. “Healthcare worker” OR “Health Professional”
4. Ethiopia
5. 1 AND 2
6. 5 AND 3 AND 4

Full searching method

((Blood) OR (Body Fluid)) AND (Exposure) AND (Occupational) AND ((Healthcare worker) OR (Health Professional)) AND (Ethiopia)

**Science Direct**

1. Occupational exposure
2. “Body fluid” OR “Blood”
3. Ethiopia
4. 1 AND 2 AND 3

Full searching method

"occupational exposure" AND ("body fluid" OR "blood") AND "Ethiopia"
