# Supplementary material for: Occupational exposures to blood and body fluids among healthcare workers in Ethiopia: a systematic review and meta-analysis
Source: Environ Health Prev Med. 2020 Oct 3;25:58. doi: 10.1186/s12199-020-00897-y (PMC7533038; doi:10.1186/s12199-020-00897-y)
Supplement: Supplementary file 4 — Additional file 4:. Sensitivity analysis for included studies of BBFs. [file 12199_2020_897_MOESM4_ESM.docx]

**Table 1: Sensitivity analysis of life time prevalence of BBFs among HCWs in Ethiopia for each study being removed at a time, 2007-2020.**

| **Name , year** | **Pooled prevalence** | **95% CI** | **I^2^ (%)** | **Q** | **p-value** |
| --- | --- | --- | --- | --- | --- |
| Zenbaba D et al., 2020 | 54.73 | 47.75-61.71 | 97.7 | 1009.41 | <0.001 |
| Geberemariyam BS, 2018 | 55.63 | 48.77-62.48 | 97.6 | 943.57 | <0.001 |
| Reda AA et al., 2010 | 56.06 | 49.59-62.52 | 97.3 | 847.27 | <0.001 |
| Geberemariyam BS, 2019 | 55.46 | 48.57-62.35 | 97.7 | 997.97 | <0.001 |
| Kaweti and Abegaz, 2014 | 56.09 | 49.69-62.50 | 97.2 | 829.08 | <0.001 |
| Amerga and Mekonnen, 2018 | 55.26 | 48.31-62.22 | 97.7 | 1007.01 | <0.001 |
| Tadesse M et al., 2016 | 54.15 | 47.46-60.83 | 97.4 | 884.31 | <0.001 |
| Yenesew and Fekadu, 2014 | 54.06 | 47.34-60.78 | 97.5 | 931.33 | <0.001 |
| Yakob E et al., 2015 | 55.34 | 48.47-62.21 | 97.7 | 1009.91 | <0.001 |
| Mengesha and Yirsaw, 2014 | 54.32 | 47.47-61.16 | 97.7 | 995.56 | <0.001 |
| Asmr Y et al., 2019 | 55.69 | 48.86-62.52 | 97.7 | 997.35 | <0.001 |
| Gebremariam AA, 2019 | 53.85 | 47.37-60.32 | 97.3 | 854.57 | <0.001 |
| Desalegn Z et al., 2015 | 54.20 | 47.39-61.02 | 97.6 | 970.69 | <0.001 |
| Desta B, 2017 | 54.86 | 47.97-61.75 | 97.7 | 1014.46 | <0.001 |
| Hebo HJ et al., 2019 | 54.74 | 47.81-61.66 | 97.7 | 1011.93 | <0.001 |
| Yasin J et al., 2019 | 54.80 | 47.85-61.75 | 97.7 | 1012.26 | <0.001 |
| Abeje and Azage, 2015 | 54.35 | 47.47-61.22 | 97.6 | 975.94 | <0.001 |
| Sahiledengle B et al., 2018 | 55.59 | 48.71-62.47 | 97.6 | 955.44 | <0.001 |
| Tebeje and Hailu, 2010 | 54.86 | 47.92-61.80 | 97.7 | 1047.13 | <0.001 |
| Akalu GT et al., 2016 | 54.85 | 47.89-61.81 | 97.7 | 1013.90 | <0.001 |
| Belachew YB et al., 2017 | 54.63 | 47.69-61.57 | 97.7 | 1005.74 | <0.001 |
| Damta M, 2007 | 55.33 | 48.39-62.27 | 97.7 | 1003.43 | <0.001 |
| Atlaw WD, 2013 | 54.46 | 47.56-61.37 | 97.7 | 995.65 | <0.001 |
| Abreha N, 2018 | 54.87 | 48.00-61.75 | 97.7 | 1014.56 | <0.001 |
| Alemu B, 2014 | 55.53 | 48.66-62.40 | 97.7 | 995.00 | <0.001 |

**Table 2: Sensitivity analysis of 12 month prevalence of BBFs among HCWs in Ethiopia for each study being removed at a time, 2007-2020.**

| **Name , year** | **Pooled prevalence** | **95% CI** | **I^2^ (%)** | **Q** | **p-value** |
| --- | --- | --- | --- | --- | --- |
| Zenbaba D et al., 2020 | 44.22 | 36.61-51.82 | 98.0 | 1055.79 | <0.001 |
| Reda AA et al., 2010 | 45.35 | 38.10-52.60 | 97.7 | 919.87 | <0.001 |
| Geberemariyam BS, 2019 | 44.93 | 37.41-52.45 | 98.0 | 1040.20 | <0.001 |
| Amare Z et al., 2018 | 44.52 | 37.00-52.05 | 98.1 | 1058.06 | <0.001 |
| Gebresilassie A et al., 2014 | 43.51 | 36.14-50.87 | 97.9 | 977.83 | <0.001 |
| Amerga and Mekonnen, 2018 | 44.43 | 36.83-52.03 | 98.0 | 1058.66 | <0.001 |
| Yenesew and Fekadu, 2014 | 43.25 | 36.00-50.50 | 97.8 | 965.39 | <0.001 |
| Jemaneh L, 2014 | 44.97 | 37.50-52.44 | 98.0 | 1046.51 | <0.001 |
| Tesfay and Habtewold, 2014 | 43.68 | 36.23-51.13 | 98.0 | 1033.70 | <0.001 |
| Beyera and Beyen, 2014 | 44.42 | 36.80-52.04 | 98.0 | 1058.68 | <0.001 |
| Yallew WW, 2017 | 43.67 | 36.21-51.13 | 97.9 | 1013.68 | <0.001 |
| Hebo HJ et al., 2019 | 44.30 | 36.76-51.84 | 98.0 | 1058.20 | <0.001 |
| Yasin J et al., 2019 | 44.48 | 36.92-52.05 | 98.0 | 1058.34 | <0.001 |
| Sahiledengle B et al., 2018 | 45.52 | 38.78-52.25 | 97.3 | 765.48 | <0.001 |
| Yimechew Z et al., 2013 | 43.42 | 36.05-50.79 | 97.9 | 1007.08 | <0.001 |
| Damta M, 2007 | 45.35 | 38.03-52.66 | 97.8 | 960.79 | <0.001 |
| Atlaw WD, 2013 | 44.73 | 37.18-52.29 | 98.0 | 1051.65 | <0.001 |
| Gebreselassie FT, 2009 | 43.24 | 35.88-50.60 | 98.0 | 1024.86 | <0.001 |
| Alemayehu T et al., 2016 | 44.26 | 36.72-51.81 | 98.0 | 1057.71 | <0.001 |
| Tadesse M et al., 2016 | 43.24 | 36.21-50.27 | 97.6 | 871.29 | <0.001 |
| Girmaye E et al., 2018 | 44.69 | 37.15-52.23 | 98.0 | 1054.24 | <0.001 |
| Shiferaw Y et al., 2012 | 43.21 | 35.87-50.56 | 97.9 | 1016.78 | <0.001 |
| Kaweti and Abegaz, 2014 | 44.17 | 36.53-51.80 | 98.0 | 1052.81 | <0.001 |
